# Development and validation of a risk prediction model for postoperative pneumonia in adult patients undergoing Stanford type A acute aortic dissection surgery: a case control study

**DOI:** 10.1186/s13019-022-01769-y

**Published:** 2022-02-23

**Authors:** Dashuai Wang, Xiaerzhati Abuduaini, Xiaofan Huang, Hongfei Wang, Xing Chen, Sheng Le, Manhua Chen, Xinling Du

**Affiliations:** 1 Department of Cardiovascular Surgery, Union Hospital, Tongji Medical College, Huazhong University of Science and Technology, Wuhan Jiefang Road, No. 1277, Wuhan, 430022 China; 2 Department of Cardiology, The Central Hospital of Wuhan, Tongji Medical College, Huazhong University of Science and Technology, Wuhan, China

**Keywords:** Pneumonia, Stanford type A aortic dissection, Risk factor, Prediction model, Nomogram

## Abstract

**Background:**

Pneumonia is a common complication after Stanford type A acute aortic dissection surgery (AADS) and contributes significantly to morbidity, mortality, and length of stay. The purpose of this study was to identify independent risk factors associated with pneumonia after AADS and to develop and validate a risk prediction model.

**Methods:**

Adults undergoing AADS between 2016 and 2019 were identified in a single-institution database. Patients were randomly divided into training and validation sets at a ratio of 2:1. Preoperative and intraoperative variables were included for analysis. A multivariate logistic regression model was constructed using significant variables from univariate analysis in the training set. A nomogram was constructed for clinical utility and the model was validated in an independent dataset.

**Results:**

Postoperative pneumonia developed in 170 of 492 patients (34.6%). In the training set, multivariate analysis identified seven independent predictors for pneumonia after AADS including age, smoking history, chronic obstructive pulmonary disease, renal insufficiency, leucocytosis, low platelet count, and intraoperative transfusion of red blood cells. The model demonstrated good calibration (Hosmer–Lemeshow χ^2^ = 3.31, *P* = 0.91) and discrimination (C-index = 0.77) in the training set. The model was also well calibrated (Hosmer–Lemeshow χ^2^ = 5.73, *P* = 0.68) and showed reliable discriminatory ability (C-index = 0.78) in the validation set. By visual inspection, the calibrations were good in both the training and validation sets.

**Conclusion:**

We developed and validated a risk prediction model for pneumonia after AADS. The model may have clinical utility in individualized risk evaluation and perioperative management.

## Introduction

Postoperative pneumonia (POP) is one of the most frequent complications after cardiac surgery, associated with increased morbidity, mortality, and treatment costs [1, 2]. The incidence of POP reported in the literature varies greatly from 2.1% to 24.2% [3].

Numerous studies have been conducted to identify risk factors for the development of POP after cardiac surgery [3]. Some predictors have been widely reported such as age, poor cardiac function, and chronic lung disease. Several risk models and scores incorporating those risk factors have also been established to predict the occurrence of POP following cardiac surgery [4–7]. As the basic characteristics of patients and surgical procedures have changed over the years, some models have also been updated [8]. However, most of the published studies have been completed in patients undergoing valve surgery, coronary artery bypass grafting, and multiple surgical types. To our knowledge, studies that designed to identify risk factors for POP in patients undergoing Stanford type A acute aortic dissection surgery (AADS) are still lacking. The establishment of a convincing prediction model used to assess the risk of POP after AADS is still in urgent need.

The aim of this observational study was to identify significant predictors for the occurrence of POP following AADS in adult patients and to develop as well as validate a risk prediction model.

## Methods

### Ethical statement

This study was conducted in accordance with the ethics statement of the Declaration of Helsinki, and was approved by The Ethics Committee of Tongji Medical College of Huazhong University of Science and Technology (IORG No. IORG0003571). Written informed consent was waived because of the retrospective, observational nature of this study.

### Study population

We conducted a single-center study that included consecutive adult patients (age ≥ 18 years) undergoing AADS within 48 h from admission between January 2016 and December 2019. AADS was diagnosed using enhanced computed tomography scanning and echocardiography. The exclusion criteria were shown as follows: (1) prior pneumonia within two weeks before surgery, (2) death intraoperatively or within 48 h after surgery, and (3) records with missing data.

### Data collection and variables

Clinical data collection was performed using the electronic medical records management system of the hospital. Preoperative variables and intraoperative variables were evaluated in this study. Demographic variables included age, sex, weight, height, body mass index, smoking history, and drinking history. Comorbidities and medical histories included hypertension, diabetes mellitus, cerebrovascular disease, peripheral vascular disease, atrial fibrillation, New York Heart Association class, left ventricular ejection fraction, chronic obstructive pulmonary disease (COPD), renal insufficiency, gastrointestinal tract disease, pulmonary artery hypertension, pericardial effusion, general surgical history, and cardiac surgery history. Preoperative data of routine laboratory blood tests were also available. Operative variables included cardiopulmonary bypass time, aortic cross clamp time, and transfusion of red blood cells. Postoperative deaths were also collected and compared.

### Definitions of important variables

Body mass index was calculated as weight in kilograms divided by the square of the height in meters. Smoking history was defined as current smoking. Drinking history was defined as current drinking. COPD was defined as FEV1/FVC ≤ 0.7. Hypertension was defined as previous diagnosis of hypertension, blood pressure ≥ 140/90 mmHg, or use of antihypertensive medication. Diabetes mellitus was defined as previous diagnosis of diabetes mellitus, fasting glucose ≥ 7.0 mmol/L, random glucose ≥ 11.1 mmol/L, or use of diabetic medication. Renal insufficiency was defined as previous diagnosis of renal insufficiency or serum creatinine > 110 μmol/L.

### Diagnosis

POP was diagnosed according to previously published criteria [9]. In this study, POP was diagnosed when a X-ray showed new and/or progressive pulmonary infiltrates combined with at least two of the following criteria: (1) fever (> 38 ℃) without other confirmed aetiology, (2) leucocytosis (> 12 × 10^9^/L) or leucopenia (< 4 × 10^9^/L), and (3) purulent secretions.

### Statistical analysis

Continuous variables were presented as means ± standard deviations or medians with inter-quartile range. Categorical variables were presented as counts with percentages. For univariate analysis, continuous data were analyzed by two-sample independent *t*-test or Mann–Whitney U test, and categorical data were analyzed by chi-square test or Fisher’s exact test. The initial dataset was randomly divided into the training set and validation set by 2:1 ratio. The former was used to develop the model, and the latter was used to validate the model. In the training set, univariate analysis was first applied to screen potential risk factors for POP after AADS. Variables with *P* < 0.1 were further enrolled into the multivariate logistic regression analysis to identify independent risk factors. A nomogram was constructed on the basis of the multivariate logistic regression model to predict the risk of POP following AADS.

Discrimination of the prediction model in the training set was evaluated by C-index or the area under the receiver operating characteristic (ROC) curve (AUC). Calibration was visually assessed by a smoothed nonparametric calibration curve and a fitted logistic calibration curve. The Hosmer–Lemeshow goodness-of-fit statistic was also calculated to test the lack-of-fit. Internal validation was evaluated by the bootstrap method using 1000 replications. External validation was performed in the validation set with the discrimination and calibration being similarly assessed. Comparison of the AUC between two groups was performed using the Delong method [10]. Clinical usefulness was assessed by decision curve analysis.

A two-tailed *P* value of less than 0.05 was considered statistically significant. Statistical analyses were performed with R software (version 4.0.3, https://www.R-project.org/).

## Results

**Fig. 1:**
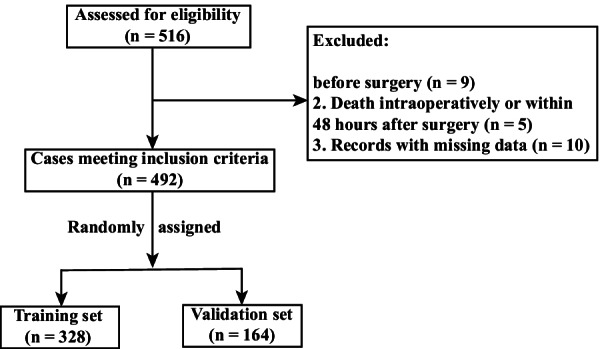
Flow chart of the study

Among the 516 adult patients who underwent AADS within 48 h from admission, nine has preoperative pneumonia, five died intraoperatively or within 48 h after surgery, and ten had missing data. The remaining 492 patients undergoing AADS fulfilled the inclusion criteria and were incorporated in the present study (Fig. 1). POP developed in 170 of the 492 patients (34.6%). Overall mortality after AADS was 9.96% (49/492). Patients with POP showed a significantly higher mortality compared with patients without POP (24.1% vs 2.5%; odds ratio [OR], 12.48; 95% confidence interval [CI], 5.69–27.34; *P* < 0.001).

The average age of the included patients was 49.6 ± 11.3 years; 75.6% were men. The most common comorbidity was hypertension, which was present in 68.1% of the patients. Patients with smoking history made up 43.9%, drinking history 35.8%, renal insufficiency 35.2%, cerebrovascular disease 17.9%, peripheral vascular disease 13.6%, cardiac surgery history 6.5%, COPD 4.9%, diabetes mellitus 4.3%. The average duration of cardiopulmonary bypass was 211 (175, 257) minutes, and the average volume of intraoperative transfusion of red blood cells were 7.5 (6, 9) units. Patients assigned to the training and validation sets were similar with respect to baseline characteristics, comorbidities and operative variables (Table 1). The incidence of POP in the two sets was 33.8% and 36.0%, respectively (*P* = 0.64).

**Table 1:** Comparison of characteristics between the training and validation sets

Characteristic	Training set n = 328 (%)	Validation set n = 164 (%)	*P* value
**Demographics**			
Age (years)	49.4 ± 11.2	51.2 ± 11.6	0.426
Male	254 (77.4)	118 (72.0)	0.181
Body mass index (kg/m^2^)	25.5 ± 3.8	25.1 ± 3.4	0.289
Smoking history	138 (42.1)	78 (47.6)	0.248
Drinking history	109 (33.2)	67 (40.9)	0.118
Underlying conditions			
Hypertension	221 (67.4)	114 (69.5)	0.632
Diabetes mellitus	15 (4.6)	6 (3.7)	0.636
Chronic obstructive pulmonary disease	16 (4.9)	8 (4.9)	1.000
Cerebrovascular disease	53 (16.2)	35 (21.3)	0.157
Peripheral vascular disease	43 (13.1)	24 (14.6)	0.642
Renal insufficiency	118 (36.0)	55 (33.5)	0.593
Gastrointestinal tract disease	26 (7.9)	16 (9.8)	0.494
Atrial fibrillation	2 (0.6)	2 (1.2)	0.478
General surgery history	69 (21.0)	32 (19.5)	0.693
Heart surgery history	25 (7.6)	7 (4.3)	0.155
New York Heart Association class			0.405
I	211 (64.3)	114 (69.5)	
II	85 (25.9)	41 (25.0)	
III	26 (7.9)	7 (4.3)	
IV	6 (1.8)	2 (1.2)	
Pulmonary artery hypertension	9 (2.7)	5 (3.0)	0.848
Pericardial effusion	97 (29.6)	36 (22.0)	0.092
Left ventricular ejection fraction (%)	62 (60, 65)	62 (60, 65)	0.892
Laboratory values			
White blood cell count (× 10^9^/L)	10.4 (7.4, 12.6)	9.7 (7.5, 13.0)	0.713
Red blood cell count (× 10^12^/L)	4.2 (3.8, 4.6)	4.2 (3.8, 4.6)	0.484
Hemoglobin (g/l)	129 (113, 139)	127 (114, 140)	0.650
Platelet count (× 10^9^/L)	160 (127, 208)	159 (122, 191)	0.174
Serum creatinine (μmol/L)	81.4 (65.9, 111.6)	78.1 (65.5, 112.8)	0.306
Serum albumin (g/L)	37.7 ± 4.4	37.6 ± 4.7	0.886
Operative variables			
Cardiopulmonary bypass time (minutes)	211 (174, 257)	210 (175, 258)	0.924
Aortic cross clamp time (minutes)	118 (96, 147)	121 (96, 149)	0.380
Transfusion of red blood cells (units)	7.5 (6.0, 8.5)	7.0 (6.0, 9.0)	0.865

### Derivation of the prediction model

**Fig. 2:**
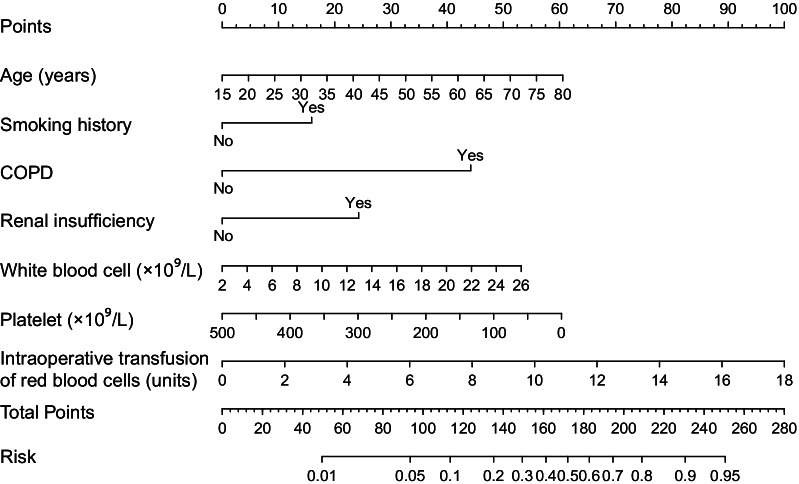
Nomogram for the prediction of POP in patients undergoing AADS. *AADS* Stanford type A acute aortic dissection surgery; *COPD* chronic obstructive pulmonary disease; *POP* postoperative pneumonia

Univariate analysis of potential risk factors for POP after AADS in the training set is displayed in Table 2. Before the construction of a multivariate model, collinearity diagnostics were performed. Significant univariate predictors that were further entered into the multivariate logistic regression model included age, sex, smoking history, COPD, renal insufficiency, white blood cell count, platelet count, cardiopulmonary bypass time, and intraoperative transfusion of red blood cells. Seven independent variables were identified in the final multivariate model by stepwise forward selection including age, smoking history, COPD, renal insufficiency, white blood cell count, platelet count, and intraoperative transfusion of red blood cells (Table 3). A nomogram was then established based on the multivariate logistic regression model to predict the risk of POP after AADS (Fig. 2). Regression coefficients of all variables were scaled to points within the range of 0–100. The relative importance of predictors can be reflected by their points.

**Table 2:** Univariate analysis of risk factors for POP after AADS in the training set

Characteristic	Without POP n = 217 (%)	With POP n = 111 (%)	χ^2^/Z/t	*P* value
**Demographics**				
Age (years)	48.0 ± 11.4	52.0 ± 10.3	3.067	0.002
Male	161 (74.2)	93 (83.8)	3.866	0.049
Body mass index (kg/m^2^)	25.3 ± 3.7	25.8 ± 4.0	1.254	0.211
Smoking history	80 (36.9)	58 (52.3)	7.133	0.008
Drinking history	72 (33.2)	37 (33.3)	0.001	0.978
Underlying conditions				
Hypertension	145 (66.8)	76 (68.5)	0.091	0.763
Diabetes mellitus	9 (4.1)	6 (5.4)	0.266	0.606
Chronic obstructive pulmonary disease	5 (2.3)	11 (9.9)	9.155	0.002
Cerebrovascular disease	30 (13.8)	23 (20.7)	2.578	0.108
Peripheral vascular disease	25 (11.5)	18 (16.2)	1.421	0.233
Renal insufficiency	58 (26.7)	60 (54.1)	23.807	< 0.001
Gastrointestinal tract disease	18 (8.3)	8 (7.2)	0.119	0.730
Atrial fibrillation	1 (0.5)	1 (0.9)	0.235	0.628
General surgery history	45 (20.7)	24 (21.6)	0.035	0.852
Heart surgery history	16 (7.4)	9 (8.1)	0.056	0.812
New York Heart Association class			4.230	0.238
I	137 (63.1)	74 (66.7)		
II	61 (28.1)	24 (21.6)		
III	17 (7.8)	9 (8.1)		
IV	2 (0.9)	4 (3.6)		
Pulmonary artery hypertension	7 (3.2)	2 (1.8)	0.558	0.455
Pericardial effusion	68 (31.3)	29 (26.1)	0.957	0.328
Left ventricular ejection fraction (%)	62 (60, 65)	62 (60, 65)	0.343	0.732
Laboratory values				
White blood cell count (× 10^9^/L)	9.8 (7.3, 12.2)	11.1 (8.2, 14.1)	2.614	0.009
Red blood cell count (× 10^12^/L)	4.2 (3.8, 4.6)	4.2 (3.7, 4.5)	0.126	0.900
Hemoglobin (g/l)	128 (112, 138)	130 (114, 139)	0.653	0.514
Platelet count (× 10^9^/L)	171 (133, 218)	143 (120, 185)	3.409	0.001
Serum creatinine (μmol/L)	78.5 (66.0, 104.6)	88.2 (65.8, 129.5)	2.218	0.027
Serum albumin (g/L)	37.9 ± 4.3	37.3 ± 4.5	1.183	0.238
Cardiopulmonary bypass time (minutes)	206 (169, 247)	231 (186, 275)	3.027	0.002
Aortic cross clamp time (minutes)	117 (94, 142)	126 (102, 160)	2.177	0.029
Transfusion of red blood cells (units)	6.5 (6, 8.5)	8.5 (6.5, 10.5)	4.749	< 0.001

*AADS* Stanford type A acute aortic dissection surgery; *POP* postoperative pneumonia

**Table 3:** Multivariate analysis of independent risk factors for POP after AADS

Characteristic	Coefficient	OR (95% CI)	*P* value
Age (years)	0.035	1.036 (1.009–1.062)	0.007
Smoking history	0.597	1.816 (1.073–3.073)	0.026
Renal insufficiency	0.910	2.484 (1.441–4.284)	0.001
Chronic obstructive pulmonary disease	1.659	5.252 (1.558–17.710)	0.007
White blood cell count (× 10^9^/L)	0.083	1.087 (1.009–1.170)	0.027
Platelet count (× 10^9^/L)	− 0.005	0.995 (0.991–0.999)	0.036
Transfusion of red blood cells (units)	0.209	1.232 (1.105–1.373)	< 0.001
Intercept	− 4.884	0.008	< 0.001

*AADS* Stanford type A acute aortic dissection surgery; *CI* confidence interval; *OR* odds ratio; *POP* postoperative pneumonia

By summing the points of all variables, we can easily predict the probability of POP after AADS in a specific patient. Older patients who had smoking history, COPD, renal insufficiency, higher white blood cell count, lower platelet count and more intraoperative transfusion of red blood cells had higher points and thus higher risk of POP. The probability of POP in patients undergoing AADS predicted by our nomogram ranged from 0.01 to 0.95.

To better serve the needs of modern clinical work, we further expanded our model to an easy-to-use risk calculator which is available online (https://pneumonia-after-aads.shinyapps.io/dynnomapp/). When using this calculator, press the “Quit” button in the bottom-left corner to reload the application first. Fill in the information of a patient and click the “Predict” button, the predicted risk of pneumonia after AADS was presented in the “Graphical summary” area on the right. The information of the corresponding patient and the model are presented in the “Numerical summary” and “Model summary” module.

### Model assessment and validation

**Fig. 3:**
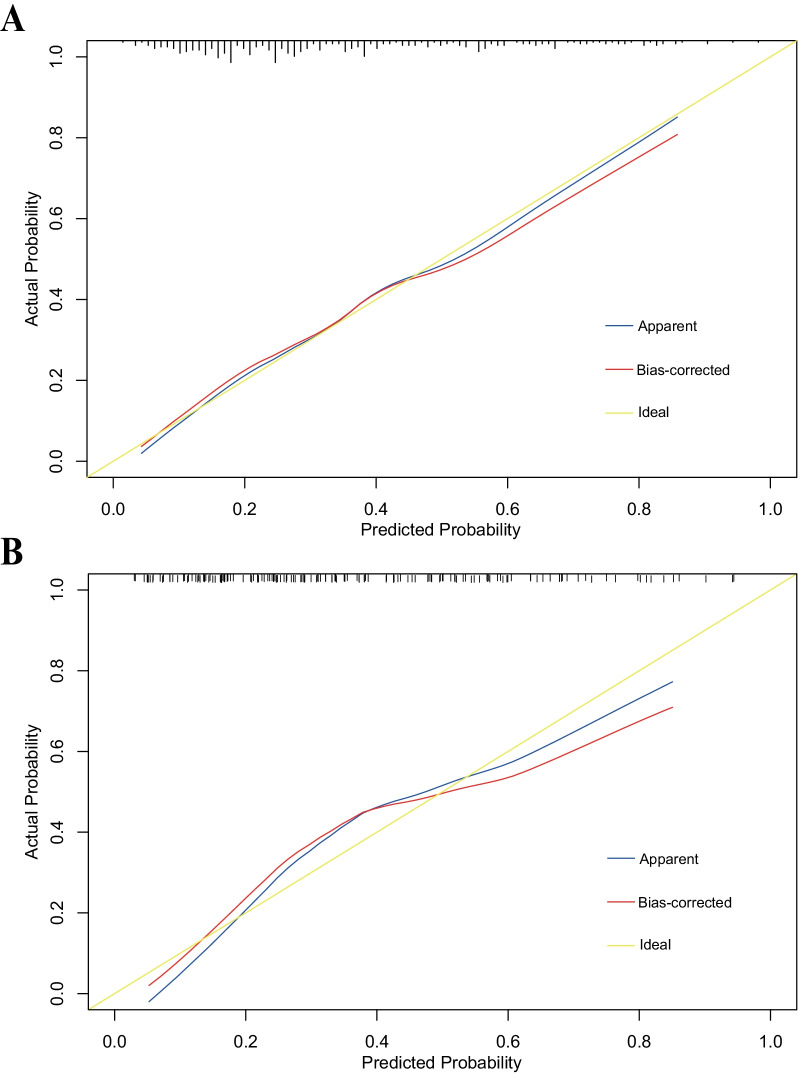
Calibration plots of the nomogram for the probability of POP after AADS in the training set (**A**) and the validation set (**B**). *AADS* Stanford type A acute aortic dissection surgery; *POP* postoperative pneumonia

**Fig. 4:**
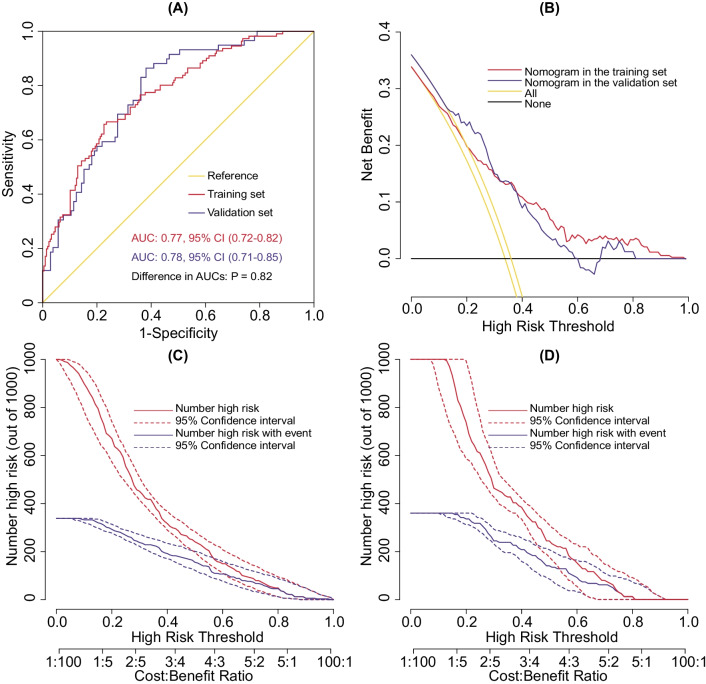
The ROC curves and decision curve analysis in the training and validation sets. ROC curves and comparison between the two AUCs (**A**), decision curves in the two sets (**B**), clinical impact curves in the training set (**C**) and the validation set (**D**). *AUC* area under the receiver operating characteristic curve; *CI* confidence interval; *ROC* receiver operating characteristic

To test the performance of the nomogram, internal validation with a calibration plot was performed using 1000 bootstrap resamples in the training set (Fig. 3A). The nomogram was well calibrated by both visual inspection and the goodness-of-fit test (Hosmer–Lemeshow χ^2^ = 3.31, *P* = 0.91). In the validation set, the calibration plot of predicted probabilities against observed POP rates showed a high level of consistency, indicating good calibration (Hosmer–Lemeshow χ^2^ = 5.73, *P* = 0.68; Fig. 3B). To further evaluate the effectiveness of the nomogram at predicting the risk of POP after AADS, the ROC curves in both the training and validation sets were drawn (Fig. 4A). The AUC was 0.77 (95% CI, 0.72–0.82) in the training set, demonstrating reasonable discrimination. When the algorithm was applied to the independent validation set, the discrimination was maintained (AUC = 0.78, 95% CI, 0.71–0.85). No significant difference was found between the training and validation sets (*P* = 0.82). The results confirmed the utility of the nomogram in predicting the occurrence of POP after AADS. To assess the clinical usefulness of the nomogram, we conducted decision curve analysis and decision and clinical impact curves were depicted. The decision curves indicated that compared to the “treat-all” and “treat-none” strategies, more clinical net benefits could be obtained across almost the whole ranges by the nomogram in both the training and validation sets (Fig. 4B). The clinical impact curves also showed that the model had reasonable and remarkable power in clinical usefulness for prediction (Fig. 4C, D).

### Clinical outcomes

The overall mortality in this study was 9.96% (49/492). However, the mortality was significantly higher in patients with POP compared to those without POP (2.5% versus 24.1%, P < 0.001). Significantly higher rates of reintubation, tracheostomy, and readmission to ICU were also observed in patients with POP. In addition, the lengths of mechanical ventilation, ICU stay and hospital stay were also significantly longer in POP group. Details of comparison are presented in Table 4.

**Table 4:** Clinical outcomes in patients with and without POP after AADS

Variables	Without POP n = 322 (%)	With POP n = 170 (%)	χ^2^/Z	*P* value
Mechanical ventilation (hours)	45.7 (35.3, 66.3)	111.1 (79.0, 185.0)	13.051	< 0.001
Reintubation	10 (3.1)	62 (36.5)	99.145	< 0.001
Tracheostomy	5 (1.6)	50 (29.4)	86.967	< 0.001
Readmission to ICU	18 (5.6)	26 (15.3)	12.866	< 0.001
ICU stay (hours)	114.4 (88.7, 159.1)	301.9 (202.0, 454.5)	14.232	< 0.001
Hospital stay (days)	19 (16, 23)	28 (22, 38)	9.570	< 0.001
Mortality	8 (2.5)	41 (24.1)	58.064	< 0.001

*AADS* Stanford type A acute aortic dissection surgery; *ICU* intensive care unit; *POP* postoperative pneumonia

## Discussion

In this study, we used data from 492 patients undergoing AADS at our institution to develop and validate a a prediction model for POP. The overall mortality was 9.96% in this study, however, the rate was significantly higher in patients with POP. Other clinical outcomes were also significantly poorer in patients with POP. By multivariate logistic regression analysis, seven independent predictors associated with the occurrence of POP were identified including age, smoking history, COPD, renal insufficiency, white blood cell count, platelet count, and intraoperative transfusion of red blood cells. A nomogram for predicting the probability of POP after AADS was then constructed. The nomogram may have particular clinical utility as it can predict a wide range of POP risk (1–95%) and is easy to calculate.

Stanford type A aortic dissection is an aggressive and life-threatening cardiac disease and emergency surgery is the primary treatment strategy [11]. Complications after AADS are more frequent than other cardiovascular surgery due to the complicated operation with longer operation time and greater trauma. The incidence of POP in this study was far higher than that previously reported in patients after cardiac surgery. The mortality of patients with POP was much higher than that of patients without POP, consistent with other cardiac surgeries. The high mortality and morbidity rates in patients with POP after AADS emphasized the need of identifying independent risk factors.

Many studies have been conducted to identify predictors for POP after cardiac surgery and several prediction models have been published [4–6]. However, to our knowledge, the work that we report represents the first validated clinical prediction model specific to POP after AADS. Our model demonstrated good discrimination and calibration, and was well validated. No significant difference was found between the training set and the validation set regarding the discriminatory ability. This result minimized the potential of overfitting and improved the reliability of the model to have a good generalization ability.

Although independent risk factors for POP after cardiac surgery identified in different studies vary considerably, advanced age as a predictor for POP has been extensively reported in the literature [4–6], which was also identified in our analysis. The mean age of the included patients in this study was approximately 50 years, obviously lower than that of patients undergoing cardiac surgery in previously published studies. Even so, the incidence of POP after AADS increased significantly with age in the present study. Renal insufficiency is another frequently reported predictor for POP after cardiac surgery [4, 7]. Because of the particularity of Stanford type A aortic dissection, the proportion of patients who had chronic or acute renal insufficiency exceeded one-third in this study, much higher than that reported in other cardiac procedures. Nonetheless, similar to the results of published studies, a significant difference in the incidence of POP was observed between patients with and without renal insufficiency in our analysis. Preoperative leucocytosis was also found to be an independent risk factor for POP after AADS. An elevated white blood cell count has been observed in patients with aortic dissection in previous studies and was associated with adverse events [12]. The elevation of the white blood cell count may associate with the acute phase systemic inflammatory response [13].

In addition to predicting the individualized risk of POP after AADS, the model may have clinical utility in the reduction of POP and preventative measures. Smoking has been identified as a preoperative predictor for POP following cardiac surgery in many studies, which may enhance pathogen colonization of the airway [14]. Kinlin and colleagues found that patients who had smoking history had 1.79-fold increased odds of POP [7], almost identical to the results reported in this study. Al-Sarraf and colleagues reported that compared with patients who quitted smoking for more than four weeks, current smokers had higher rates of postoperative pulmonary complications after cardiac surgery [15]. Overall, smoking cessation should not only be encouraged in patients with high risk, but also in general population for long-term primary prevention or general health maintenance [14].

COPD was identified as the most important risk factor for the occurrence of POP after AADS in this study. This was in agreement with the results reported in the literature [3, 4]. Whitman and colleagues reported that the risk of POP after cardiac surgery increased with the severity of chronic lung disease [5]. A systematic review and meta-analysis by Furukawa and colleagues found that preoperative inspiratory muscle training was associated with a reduction of POP and duration of hospital stay in adult patients undergoing cardiac surgery [16]. Preoperative optimization by respiratory physiotherapy may be a good option for patients undergoing elective cardiac surgery. However, it remains to be future investigated to what extent these findings can be translated into clinical practice of AADS.

Preoperative platelet count was another predictor associated with the development of POP after AADS in our analysis, in agreement with a recent study [17]. These researchers found that low platelet count was an independent risk factor of POP with a specific predictive power in patients after AADS [17]. Their results revealed that a 10 × 10^9^/L decrease of platelet count was related to a 7% increase in the risk of POP. Although the association between a low platelet count and POP in patients after AADS has been reported previously, the underlying mechanism of their relationship has not been well understood at present. One possible explanation is that the reduction of platelet count is associated with platelet activation that may secrete pro-inflammatory cytokines and chemokines to initiate inflammatory responses [18]. These pro-inflammatory molecules may exacerbate neutrophil rolling, adhesion and recruitment, promoting lung inflammation and increasing the severity of pneumonia [19]. Another possible explanation is that low platelet count could account for impaired coagulation and increased bleeding, associated with more blood transfusions. However, transfusion itself is linked with POP and other postoperative adverse outcomes. Gelijns and colleagues reported that platelet transfusion was protective (hazard ratio = 0.49), but each unit of red blood cells transfused increased the risk (hazard ratio = 1.16) of POP in patients after cardiac surgery [6].

Not surprisingly, intraoperative transfusion of red blood cells was identified as a significant predictor for POP in patients undergoing AADS. A dose–effect relationship was observed in this study. Although blood transfusions can be lifesaving, growing evidence indicates that this therapy may associate with higher mortality rates and serious adverse effects [20]. A prospective multicenter study found that patients who received blood transfusion had 3.4-fold increased odds of POP after cardiac surgery [21]. The risk of POP increased significantly with each unit of transfused red blood cells. Another study conducted in patients undergoing coronary artery bypass grafting reported that limited blood transfusion could reduce the risk of POP, mortality, and health care costs [22]. The association between POP and blood transfusion can be partially explained by changes of immune function [23, 24]. The length of blood storage might also affect the occurrence of POP [25]. A restrictive transfusion strategy has been recommended in clinical practice guidelines to reduce the incidence of POP and improve outcomes in patients undergoing cardiac surgery [26, 27].

Several predictors for POP after cardiac surgery that have been frequently reported in published studies were not identified as significant risk factors for POP after AADS in our analysis, including poor cardiac function, peripheral vascular disease, cardiac surgery history, and cardiopulmonary bypass time [1, 4, 5, 28]. The huge difference of risk factors for POP between AADS and other cardiac surgeries further revealed the particularity of AADS. Therefore, it may not be appropriate to predict the risk of POP following AADS using the existing risk prediction models which were developed for POP after other cardiac surgeries.

Prolonged mechanical ventilation has been recognized as an independent risk factor for POP in some studies [6, 28, 29]. Endotracheal intubation may damage the defense mechanism of the respiratory system and the risk of POP may increase with prolonged mechanical ventilation [30]. There is no doubt that extubation should be performed as soon as conditions permit [31]. However, we did not incorporate prolonged mechanical ventilation as a predictor into our analysis because it was a postoperative variable and was not available early. Reintubation and tracheotomy were also excluded from our analysis because of their nature of postoperative variables. In addition, diabetes mellitus, cerebrovascular disease, underweight, and preoperative anemia were also reported as independent risk factors for POP after cardiac surgery in some studies [4, 6, 7], but were not significant in this study.

Our prediction model may also play an important role in identifying and targeting high-risk patients for preventative measures. Several interventions have been shown to significantly reduce the incidence of POP, including subglottic secretion drainage [32], preoperative chlorhexidine mouthwash [33], and silver-coated endotracheal tubes [34]. Specific interventions and appropriate preventative measures targeting high-risk patients identified by our prediction model may yield substantial clinical and economic benefits.

Several limitations should be mentioned in this study. First, this is a single-center, retrospective study. Although our prediction model was well validated in another independent dataset, the small sample size may limit its generalizability. Whether it is applicable to other cardiac centers has yet to be studied. Second, some possible risk factors that may associate with the development of POP, such as severity and involved tracts of aortic dissection, were not included in our analysis. Even so, the current established nomogram model performed and validated well with regard to discrimination, calibration, and clinical utility. Third, we only analyzed the results of the patients in the hospital and did not perform long-term follow-up after discharge. Fourth, we included POP as the primary endpoint, but the severity of POP and its impact on other clinical outcomes and costs of care were not evaluated in this study.

## Conclusion

POP was prevalent in patients undergoing AADS, with high morbidity and mortality. We first developed and validated a multivariate prediction model for POP after AADS using seven independent risk factors and conducted a nomogram. The model performed well in terms of both discrimination and calibration, and was well validated in the independent validation dataset. We believe the model has good clinical utility as the included variables are easy to obtain and the risk is easy to calculate. The model can be used to inform clinician-patient decision-making through individualized risk evaluation and identification of high-risk populations. Its potential clinical applications lie in risk modification and targeted interventions to decrease the incidence of POP in patients undergoing AADS.

## Data Availability

The datasets used and analyzed during the current study are available from the corresponding author on reasonable request.

## Abbreviations

AADS: Stanford type A acute aortic dissection surgery
AUC: Area under the receiver operating characteristic curve
CI: Confidence interval
COPD: Chronic obstructive pulmonary disease
OR: Odds ratio
POP: Postoperative pneumonia
ROC: Receiver operating characteristic
